# Importin-8 Modulates Division of Apical Progenitors, Dendritogenesis and Tangential Migration During Development of Mouse Cortex

**DOI:** 10.3389/fnmol.2018.00234

**Published:** 2018-07-10

**Authors:** Gerry Nganou, Carla G. Silva, Ivan Gladwyn-Ng, Dominique Engel, Bernard Coumans, Antonio V. Delgado-Escueta, Miyabi Tanaka, Laurent Nguyen, Thierry Grisar, Laurence de Nijs, Bernard Lakaye

**Affiliations:** ^1^GIGA-Neurosciences, University of Liege, Liege, Belgium; ^2^GENESS International Consortium, Los Angeles, CA, United States; ^3^Epilepsy Genetics/Genomics Lab, Neurology and Research Services, VA Greater Los Angeles Healthcare System (VA GLAHS), University of California, Los Angeles, Los Angeles, CA, United States; ^4^MHeNS, Maastricht University, Maastricht, Netherlands

**Keywords:** karyopherin, importin-8, corticogenesis, radial migration, tangential migration, dendritogenesis, *in utero* electroporation

## Abstract

The building of the brain is a multistep process that requires the coordinate expression of thousands of genes and an intense nucleocytoplasmic transport of RNA and proteins. This transport is mediated by karyopherins that comprise importins and exportins. Here, we investigated the role of the ß-importin, importin-8 (IPO8) during mouse cerebral corticogenesis as several of its cargoes have been shown to be essential during this process. First, we showed that *Ipo8* mRNA is expressed in mouse brain at various embryonic ages with a clear signal in the sub-ventricular/ventricular zone (SVZ/VZ), the cerebral cortical plate (CP) and the ganglionic eminences. We found that acute knockdown of IPO8 in cortical progenitors reduced both their proliferation and cell cycle exit leading to the increase in apical progenitor pool without influencing the number of basal progenitors (BPs). Projection neurons ultimately reached their appropriate cerebral cortical layer, but their dendritogenesis was specifically affected, resulting in neurons with reduced dendrite complexity. IPO8 knockdown also slowed the migration of cortical interneurons. Together, our data demonstrate that IPO8 contribute to the coordination of several critical steps of cerebral cortex development. These results suggest that the impairment of IPO8 function might be associated with some diseases of neuronal migration defects.

## Introduction

The mouse cerebral cortex is comprised of excitatory projection and inhibitory GABAergic interneurons −85% and 15% respectively, which arise from different progenitor zones during development (Anderson and Vanderhaeghen, 2015). The former is generated from progenitors located in the ventricular zone (VZ) of the dorsal telencephalon. Apical radial glial cells (aRGCs, also known as apical progenitors (APs)) undergo mitosis at the apical surface of the VZ, where they initially undertake symmetric divisions to expand their population, but progressively switch to asymmetric division during the progression of corticogenesis (Chou and O’Leary, 2013; Daviaud et al., 2016). The latter process generates one self-renewing AP and either a basal progenitor (BP) or an excitatory projection neuron. These BPs (also known as subventricular zone (SVZ) progenitors) may undergo symmetric division to generate either two postmitotic projection neurons or, more rarely, two daughter BPs (reviewed in Dwyer et al., 2016). Newborn projection neurons migrate radially through the SVZ to the intermediate zone (IZ) where they adopt a multipolar morphology. Following this stage, the neurons convert to a bipolar morphology and, guided by the basal processes of aRGCs, migrate out of the IZ and pass the existing neuronal layers of the cortical plate (CP) to settle at their final destination (Luhmann et al., 2014).

Cortical GABAergic interneurons are produced in ganglionic eminences (Faux et al., 2012; Peyre et al., 2015) and migrate tangentially towards the cerebral cortex (Liu et al., 2017). During the migration process, interneurons rely upon their leading processes to sense the extra-cellular environment for guidance cues. This results in the formation of new branches that are better aligned in the direction of the source gradient. The centrosome, the Golgi apparatus and the nucleus move then forward through somal translocation, pulled and pushed by Actin, myosin and cytoplasmic dynein motor proteins (Martini and Valdeolmillos, 2010). While entering the cortex, these neurons follow different pathways to position themselves within the forming CP. Some of them enter the cerebral cortex by the upper layer and then migrate “down” to the lower region of the cortex, including the IZ, along with basal processes of aRGCs. The others reach the cerebral cortex through the IZ, then migrate “down” to VZ/SVZ before moving “up” to the CP via aRGCs’ processes (Zechel et al., 2016).

Once neurons have reached their final destination, they start to differentiate and develop neuronal processes named axons and dendrites, that will allow them to communicate with each other, locally or even over long distances, and become part of the processing network (Perry and Fainzilber, 2009).

These different steps of mouse cortical development require the highly coordinated transcriptional programs necessary for cell division and cell-fate specification, as well as the accompanying production and differentiation of new cellular compartments, and their interactions with extracellular signals (Telley et al., 2016). These developmental processes require an exceptional synchronization of the cell-intrinsic trafficking of thousands of different proteins between the cytoplasm and the nucleus for the precise control of these different events.

The cytoplasmic-nuclear shuttling of molecules is predominantly determined by karyopherins, which is a family of proteins that comprises two classes: karyopherins-α (also known as importins-α), and karyopherins-β that includes both importins-β and exportins (Mosammaparast and Pemberton, 2004). These proteins modulate the transport of RNA as well as proteins larger than 40 kDa across the nuclear membrane (Adam, 2009; Freitas and Cunha, 2009). Understandably, this protein family is an important player for the different steps of nervous system development (proliferation, migration, differentiation). Specifically, Exportin-1 interacts with Disabled-1 (DAB1), an intracellular mediator of the Reelin signaling pathway that is essential for correct neuronal positioning (Honda and Nakajima, 2006). Simply modulating the cytoplasmic and nuclear levels of DAB1 influences the radial migration of excitatory neurons (Honda and Nakajima, 2016). In addition, Neurogenin-3 is transiently exported from the nucleus to the cytoplasm of hippocampal neurons during initiation of neuronal polarity and blocking this transport changes neuronal morphology (Simon-Areces et al., 2013). In the *Drosophila*, importin-α2 is highly expressed in the larval nervous system and determines both the density of the active zones at the neuromuscular junction and axon commissure formation in the spinal cord midline (Mosca and Schwarz, 2010) whereas importin-α3 is necessary to prevent misguidance of axons in the optic lobe (Ting et al., 2007). Finally, in the Aplysia, Importin-α mediates the retrograde transport of CREB2, a transcriptional repressor that modulates synaptic plasticity, from distal processes to the nucleus (Lai et al., 2008).

Importin-8 (IPO8) is an importin-β of ~112 kDa that has been shown to be expressed in multiple tissues, including the adult brain (Søes et al., 2013) and has been reported to transport several proteins important for brain development such as Smad4, Ago2 or eIF4E for example (Yao et al., 2008; Weinmann et al., 2009; Volpon et al., 2016). However, no studies have yet investigated its potential role during cerebral corticogenesis. The above-mentioned evidences of the role of karyopherins during brain development and *Ipo8* expression in adult mouse brain prompted us to investigate whether it might have a putative role in cerebral cortex development. We showed that in mice, IPO8 plays a role in radial neuroblast migration by affecting cell cycle exit and mitosis of progenitor cells and by regulating the proportion and number of APs cells. Moreover, we found that IPO8 slowed down the migration of interneurons produced by the MGE. Finally, we demonstrated that IPO8 disturbs dendritogenesis of cortical neurons.

## Materials and Methods

### Animals

Time-mated wild type (NMRI) mice (E10.5 to E13.5) were provided by the animal facility of the University of Liege. This study was carried out in accordance with the recommendations of the guidelines of the Belgian Ministry of Agriculture in agreement with European Community Council Directive for the care and use of laboratory animals of 22 September 2010 (2010/63/EU) and approved by the local ethics committee of the University of Liège with the experimental protocols n°1324 and 1704.

### Tissue Processing

For RT-PCR and qRT-PCR, mice were sacrificed at different developmental stages (E12; E14; E18; P5 or P60). Brains were carefully dissected out from the skulls and immediately frozen. Frozen brains were grinded with a tissue homogenizer in a specific resuspension solution (see qRT-PCR).

For immunohistochemistry (IHC) or *in situ* hybridization (ISH), embryonic brains were dissected in PBS/glucose and, fixed in 4% paraformaldehyde (PFA) for 2–4 h at 4°C. For IHC in newborn brains, pups were perfused transcardially with cold 4% PFA in 0.1 M phosphate buffer (pH 7.4) after deep anesthesia with sodium pentobarbital (Nembutal, 60 mg/ml). Brains were removed and postfixed in 4% PFA for 4–6 h at 4°C. After fixation, all brains were cryoprotected in 30% (w/v) sucrose in PBS for at least 24 h at 4°C. They were then embedded in OCT medium (Tissue-Tek) for cryosectioning of 14 μm onto Superfrost slides (Thermo Scientific) for either IHC or ISH.

### Plasmids Construction

The control short hairpin RNA (shRNA) was directed against a “non-relevant” mammalian sequence (i.e., luciferase), and the shRNAs directed against mouse *Ipo8* (mIPO8) mRNA were cloned into the pCA-b-EGFPm5 silencer-3, a plasmid that co-express EGFP for easy cell detection (gift from D. Vermeren, King’s College London, UK). Their sequences are given in the supplementary data (Supplementary Figure S1). HA-tagged human IPO8 (HA-hIPO8) coding sequence and an “shRNA-resistant” form of this coding sequence (HA-hIPO8*; Supplementary Figure S2) were cloned into pCAGGS vector. This form was generated using a “g-block” fragment (IDT-DNA). All plasmids were purified using the “endoFree plasmid purification” kit (Qiagen). ISH probes correspond to two mIPO8 cDNA fragments encompassing nucleotides 2681–3253 and 3251–4000 (Acc. Num: NM_001081113). These two regions correspond to the end of the *Ipo8* coding sequence (AA 821–1010) and the beginning of the 3’UTR, respectively and show low or no identity with the mouse *Ipo7* sequence. They were amplified by RT-PCR and cloned into pGEM-T vector (Promega) for RNA probe synthesis.

### *In Situ* Hybridization (ISH)

Linearized vectors were transcribed *in vitro* using the “Digoxigenin (DIG) RNA labeling” Kit (Roche Applied Science). DIG-labeled cRNA probes were purified with MicroSpin G-50 columns (GE Healthcare). The ISH procedure was carried out according to a standard protocol (Lopez, 2014) and revelation was performed during 12 h at room temperature using NBT/BCIP (B1911, Sigma Aldrich) as substrate.

### qRT-PCR

RNA was extracted from either micro-dissected brain regions or FACS selected Neuro2A cells with the “NucleoSpin RNA” kit (Macherey-Nagel). RNA was reverse transcribed into cDNA with the use of “ProtoScript^®^ II First Strand cDNA Synthesis” kit (New England Biolabs) according to the manufacturer’s instructions. Real-time PCR was performed in triplicate with the use of the “LightCycler 480 SYBR Green I Master” kit (Roche). *Ipo8* and* Ipo7* expression were normalized to *ß-actin* and *Gapdh* using the 2^−ΔΔCt^ method as previously described (Tichopad et al., 2003). All primers were ordered from Integrated DNA Technologies (IDT) and their sequences are given in the supplementary data (Supplementary Figures S3, S4).

### Immunostaining Procedure

For immunostaining, the following primary antibodies were used: rat monoclonal antibody to GFP (B-Bridge, clone 1A, 1:1000), rabbit polyclonal antibody to GFP (Life Technologies, A6455, 1:200), rabbit polyclonal antibody to Ki67 (AbCam, ab15580, 1:250), mouse monoclonal antibody to phospho-Histone H3 (Thermo Fisher, MA5–15220, PH3, 1:500), goat polyclonal antibody to Sox2 (Santa Cruz, sc-17320, 1:250), rabbit polyclonal antibody to Tbr2 (AbCam, ab23345, 1:500), chicken polyclonal antibody to Map2 (Abcam, ab5392, 1:5000), rabbit polyclonal antibody to pericentrin (Abcam, ab4448, 1:200) or revelation kit for EdU detection (Thermo Fisher). The secondary antibodies used were: RRX-, FITC- or Cy5-conjugated antibodies to rabbit, mouse, goat or rat IgG (Jackson ImmunoResearch, 1:500). Biocytin and Rhodamine Avidin DCS were purchased from Invitrogen (B1592) and Vector Lab (A-2012), respectively.

For IHC, cryostat sections were washed one time with PBS and incubated 1 h at room temperature in blocking solution (0.3% TritonX-100, 10% normal donkey serum (Jackson ImmunoResearch) in PBS). Slides were incubated overnight at 4°C with primary antibodies and for 1 h at room temperature with secondary antibodies all diluted in blocking solution. For EdU immunostaining, after incubation with the secondary antibody, slices were incubated during 30 min with the appropriate preparation of EdU detection mix according to the protocol of kit (Thermo Fisher). Slides were washed again with PBS and mounted in Vectashield Hard Set Mounting Medium with DAPI. Images were captured using an Olympus Fluoview FV1000 confocal system equipped with an Olympus IX81 inverted microscope (Olympus or Nikon).

### Electroporation

#### *In Utero* Electroporation (IUE)

For radial migration study, *in utero* electroporation (IUE) was performed as described previously (de Nijs et al., 2012) on E14.5 timed-embryo after deep anesthesia of the pregnant mice with isoflurane in oxygen carrier (Abbot Laboratories). At different times following IUE, embryos or newborn pups were processed for tissue analyses.

For cell cycle studies, E13.5 pregnant females were subjected to IUE and injected intraperitoneally with EdU (50 mg/g body weight). For Ki67 analysis, EdU injection was done 24 h before dissection (24 h after IUE). For PH3, Sox2 or Tbr2 analysis, EdU was injected 1 h before dissection (48 h after IUE).

#### Median Ganglionic Eminence (MGE) Electroporation

Median ganglionic eminence (MGE) electroporation was adapted from Bellion et al. (2005). Briefly, a cortical feeder cell layer was prepared from E12.5 embryos. Cortex were carefully dissected in DMEM/F12 and were dissociated by 15 min of incubation at 37°C in 0.25% Trypsin/0.1% DNAse. Digestion was stopped with ovomucoid inhibitor solution and cells were resuspended in Neurobasal Medium supplemented with 1% B27 without vitamin A, 1% N2 and 2mM L-glutamine. One day later, head of E13.5 embryos were dissected in DMEM/F12 to display MGE. Electroporation (five pulses, 50V, 50 ms, 1 s interval) into MGE was done by injecting 2–4 μl of plasmid solution (2 μg/μl) supplemented with 0.05% Fast Green (Sigma) using a pulled glass micropipette and a microinjector (Femtojet, Eppendorf). After electroporation, MGE were dissected and cut in small pieces of explants in DMEM/F12. Explants were sucked with a micropipette and put on top of the cortical feeder. Explants were cultured during 1 or 2 days (at 37°C under 5% CO_2_) to allow interneurons to get out and migrate on cortical feeder. The movement of electroporated interneurons was analyzed by time-lapse imaging using a confocal microscope. Live images were taken every 5 min for 5–10 h.

### Biocytin Injection and Detection

Brains from P5 animals were rapidly isolated and immediately submerged in an ice-cold solution containing in mM: 87 NaCl, 25 NaHCO_3_, 10 D-glucose, 75 sucrose, 2.5 KCl, 1.25 NaH_2_PO_4_, 0.5 CaCl_2_ and 7 MgCl_2_, equilibrated with 95% O_2_/5% CO_2_ (320–330 mOsm). Coronal slices (300 μm-thick), cut using a vibratome (Leica VT-1200, Nussloch, Germany), were allowed to recover in a “Gibb-type” storing chamber containing standard physiological saline heated to 34°C for >60 min. After the recovery period, they were stored at room temperature. For biocytin injection, the slices were perfused with standard physiological saline containing in mM: 125 NaCl, 25 NaHCO_3_, 25 D-glucose, 2.5 KCl, 1.25 NaH_2_PO_4_, 2 CaCl_2_ and 1 MgCl_2_ (315–320 mOsm). GFP-labeled neurons were selected using an LED light source (Thorlabs, M490L3) mounted on the fluorescence port of a Zeiss Axioskop FS microscope with a GFP filter. Neurons were visualized prior to patching by switching to infrared-Dodt gradient contrast (IR-DGC). IR and fluorescence images were superimposed and observed before and during recording using an IR camera (Newvicon tube in NC-70, Dage-MTI). Patch pipettes were pulled from thick walled borosilicate glass tubing (outer diameter: 2 mm, inner diameter: 1 mm, Hilgenberg, Germany) with a horizontal puller (P-97, Sutter Instruments). Pipettes were filled with a potassium-based solution consisting of, in mM: 120 K-gluconate, 20 KCl, 2 MgCl_2_, 2 Na_2_ATP, 10 EGTA, 10 HEPES and 0.1% biocytin (pH = 7.3; osmolarity: ~302 mOsm). When filled with this solution, tip resistances were between 6 MΩ and 12 MΩ. Neurons were filled for ~20 min with biocytin during whole-cell patch-clamp recordings. An outside-out was obtained after ~20 min to terminate the biocytin filling of each neuron. Then slices were fixed for ~12 h in 4% PFA, washed and incubated overnight with Rhodamine Avidin DCS (1/1000 in TritonX-100). After washing, slices were embedded in ProLong Diamond.

### Mouse Primary Cortical Neurons Cultures

Brains of E13.5 embryos were subjected to IUE and 2 days later, the cortical region was dissected out, cells were dissociated as described for “cortical feeder preparation” and were cultured in fresh neuronal medium for 7 days. Neurons were then fixed with PFA 4% and treated for immunocytochemistry.

### Quantitative Analysis

To quantify neuronal migration after IUE, the cerebral cortex was divided into three sub-regions i.e., the VZ/SVZ, the IZ and the CP, on the basis of cell density visualized after DAPI staining. The number of EGFP-positive cells in each sub-region was counted in several sections from 18 to 19 independent embryos brains (from three mice).

For cell cycle analysis, cells in the VZ/SVZ and IZ were counted in slices from 18 to 19 independent embryos (from three mice). The cell cycle exit index was calculated by dividing the number of EGFP-positive cells that have exited the cell cycle (i.e., EGFP and EdU double positive, Ki67 negative) by the EGFP-labeled cells that have incorporated EdU (EGFP and EdU double positive). The mitotic index corresponds to the percentage of EGFP-positive cells in mitosis (EGFP, EdU and pH3 triple positive) to total EGFP-labeled cells that have incorporated EdU (EGFP and EdU double positive). The percentage of Sox2 or Tbr2 positive cells correspond to the number of EGFP/EdU/Sox2 or Tbr2 triple positive cells to the number of EGFP/EdU double positive.

Neuronal arborization complexity was analyzed by Sholl analysis (O’Neill et al., 2015; Wilson et al., 2017). Briefly, each 8-bit image of single neuron (for a total of 47–52 neurons from 3 different cultures for each condition *in vitro* and 60 neurons for each condition *in vivo*) was submitted to the “Sholl Analysis” plugging of ImageJ software.

For MGE analysis, four parameters (average speed, amplitude, frequency and persistence) were considered according to previous study (Tanaka et al., 2003; Bellion et al., 2005). After live imaging, acquired videos of migration were analyzed using “MTrackJ” plugging of ImageJ software. For each condition, a total of 21 neurons from three cultures were traced and analyzed.

### Statistical Analyses

All experiments and quantifications were performed blind. Values are presented as mean ± SEM. Statistical analyses were performed using one-way analysis of variance (ANOVA) followed by Dunnett’s *post hoc* test for multiple comparisons or *t-test* (GraphPad Prism software). Differences between the groups were considered significant for *p* < 0.05. Asterisks indicate the level of significance: *p < 0.05; **p < 0.01; ***p < 0.001 and ****p < 0.0001.

## Results

### *Ipo8* mRNA Expression and Distribution in Mouse Brain

**Figure 1:**
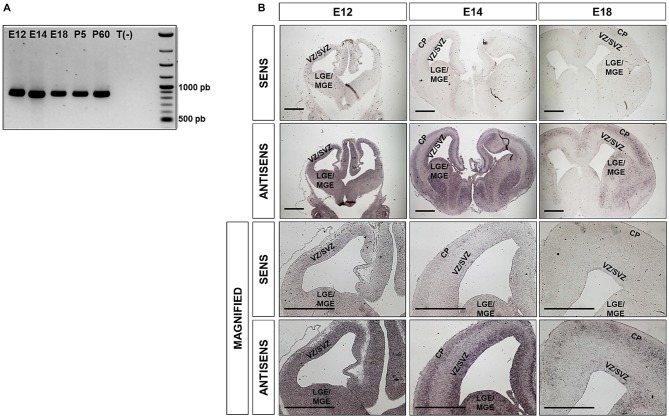
*Ipo8* mRNA is expressed in both the developing and the adult mouse brain **(A)**. RT-PCR analysis in whole brains at different embryonic (E12, E14, E18) and post-natal (P5, P60) ages. T(-) is a negative control that corresponds to a PCR performed in absence of cDNA. The expected band size is 883bp. **(B)**. *Ipo8* mRNA distribution in the developing brain visualized by *in situ* hybridization (ISH). At E12, mRNA is detected in many regions including LGE/MGE and the VZ/SVZ of the cortex. At E14 and E18, a signal is observed in the LGE/MGE, VZ/SVZ and cortical plate (CP) but tends to decrease with ages. LGE/MGE, lateral/median ganglionic eminence; VZ/SVZ, ventricular/subventricular zone (SVZ); CP, cortical plate. Scale bar: 1000 μm.

*Ipo8* has been shown to be expressed in adult brain (Søes et al., 2013) but also during development from the fertilized egg to the E10 embryo (Quan et al., 2008). However, nothing is known about its expression and localization in the developing cerebral cortex. We therefore first analyzed the mRNA levels of *Ipo8* by RT-PCR in whole brains at different milestones during embryonic development and postnatal maturation of the cerebral cortex (E12, E14, E18, P5 and P60) and detected the presence of *Ipo8* mRNA at all stages (Figure 1A).

Due to the relatively high expression of *Ipo8* in the developing mouse brain, we further investigated it spatiotemporal expression by ISH. Two different probes were used and both gave the same distribution. Only those obtained with the probe hybridizing to the 3’UTR are shown (Figure 1B). A clear and specific labeling for the transcript was detected in dorsal and ventral telencephalons of E12, E16 and E18 embryos with antisense probe when compared to the control sense probe (Figure 1B). In greater details, the mRNA was detected in the LGE/MGE and VZ/SVZ of E12 as well as in the VZ/SVZ, CP and LGE/MGE of E14 embryos (Figure 1B magnified). Similar observations were made at E18 but the labeling was less intense. These results demonstrate that *Ipo8* mRNA is expressed in specific and essential areas of the developing mouse brain.

### IPO8 Is Required for Radial Migration of Cortical Projection Neurons

**Figure 2:**
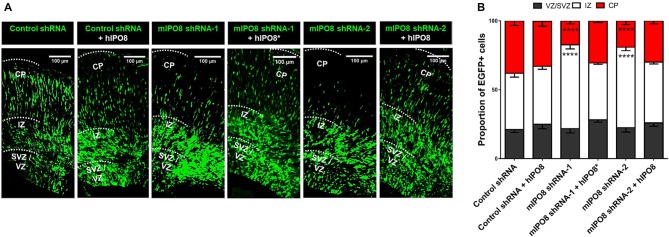
Acute *Ipo8* knockdown alters radial migration of projection neurons in the cerebral cortex. **(A)** Distribution of EGFP-positive cells 3 days after *in utero* electroporation (IUE) at E14.5. For knockdown experiments, only the plasmid co-expressing EGFP and either a control shRNA or an shRNA directed against mIPO8 (shRNA-1 and shRNA-2) was electroporated. For rescue experiments, a plasmid overexpressing a HA-tagged hIPO8* CDS along with the one producing mIPO8 shRNA-1, or one expressing HA-tagged hIPO8 CDS along with another producing mIPO8 shRNA-2 or control shRNA, were co-electroporated. **(B)** Proportion of EGFP cells in the different cortical regions. The control shRNA condition was taken as reference for statistical analysis. ****p < 0.0001. VZ/SVZ, ventricular/subventricular zone; IZ, intermediate zone; CP, cortical plate. Scale bar: 100 μm.

To investigate the role of IPO8 in cortical development and more specifically during radial migration of cortical projection neurons, we knocked down *Ipo8* expression in aRGCs by IUE at E14.5 followed by analysis at E17.5. Two shRNAs (shRNA-1 targeting the CDS and shRNA-2 targeting the 3’UTR) that efficiently decreased endogenous mIPO8 mRNA level by 70% in Neuro2A cells and a control shRNA were used (Supplementary Figure S1). In the brains subjected to IUE with the control shRNA, a vast majority of EGFP cells entered the IZ (41 ± 2.8%) or reached the CP (38 ± 3.3%; Figure 2). However, IUE with either mIPO8 shRNAs led to a significant accumulation of EGFP cells in the IZ (60 ± 3% and 58 ± 2.4%; *p* < 0.0001) at the expense of fewer EGFP cells reaching the CP (17 ± 2.1% and 19 ± 2.7%; *p* < 0.0001; Figure 2).

To validate the specificity of the suppression of *Ipo8* expression on neuronal migration, we performed rescue experiments with the human IPO8 because it exhibits 92% identity and 96% similarity at the protein level with the mouse sequence. E14.5 mouse brains were co-electroporated with either the plasmid producing mIPO8 shRNA-1 with a second encoding an shRNA-resistant form of the human protein (hIPO8*), or the plasmid producing mIPO8 shRNA-2 with a second encoding the human IPO8 (hIPO8) protein. Both of these co-electroporation conditions led to complete rescue of the migration defect, with the distribution of EGFP cells that is comparable to the control; 41% and 44% of the cells remained in the IZ for shRNA-1 and shRNA-2 (*p* > 0.5 when compared to control), whereas 31% and 30% reached the CP (*p* ≥ 0.1 when compared to control) respectively. Note that the overexpression of hIPO8 along with the control shRNA did not affect the migration (42% vs. 41% of cells in the IZ, *p* ≥ 0.5; 33% vs. 38% in the CP, *p* ≥ 0.1; Figure 2). Impairment of radial migration after *Ipo8* knockdown implicates its role in the radial migration of cortical neurons.

### IPO8 Participates in the Proliferation and Cell Cycle Exit of Apical Progenitor Cells

Since division of cortical progenitor is prior and tightly coupled to neuronal migration, we hypothesized that the failure of *Ipo8*-suppressed neurons to reach the CP may also result from impaired proliferation and/or cell cycle exit of progenitor pools.

**Figure 3:**
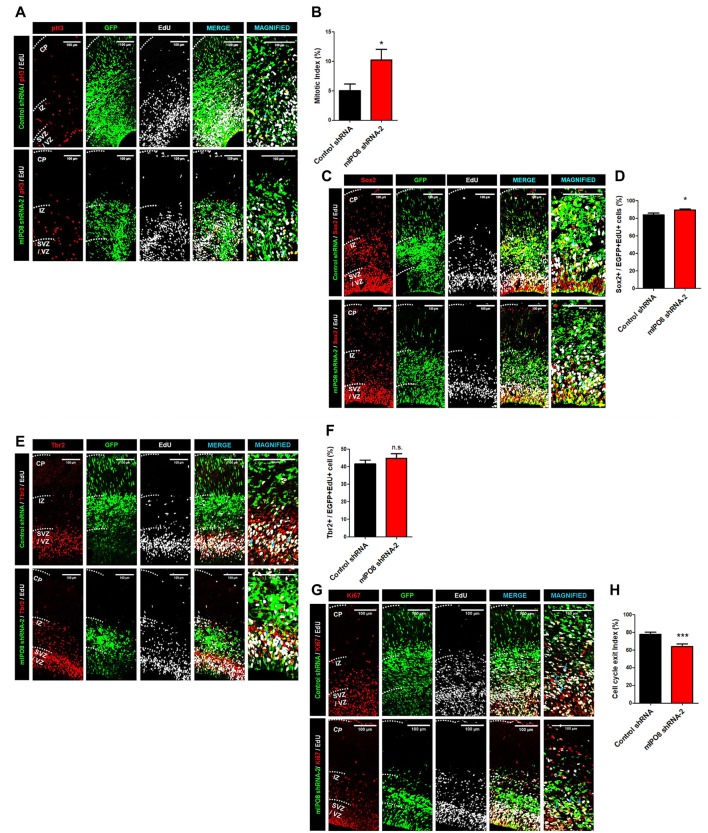
*Ipo8* knockdown affects cell proliferation and cell cycle exit of apical progenitor (AP) population. **(A,B)** Analysis of the mitotic index. IUE was performed at E13.5 with control shRNA or mIPO8 shRNA-2. Mice were injected i.p. with EdU 1 h before sacrifice at E15.5. **(A)** Immunolabeling for EGFP (green), EdU (gray) and pH3 (red), a mitotic marker. **(B)** Quantification of the mitotic index (number of EGFP+/EdU+/pH3+ cells divided by the number of EGFP+EdU+ cells). **(C,D)**. Analysis of the AP pool. The experimental conditions were the same as in **(A)**. **(C)** Immunolabeling for EGFP (green), EdU (gray) and Sox2 (red), the AP marker. **(D)** Quantification of the proportion of neurons expressing Sox2 among the EGFP+/EdU+ population. **(E,F)** Analysis of the Basal progenitor (BP) pool. The experimental conditions were the same as in **(A,B)**. **(E)** Immunolabeling for EGFP (green), EdU (gray) and Tbr2 (red), a BP marker. **(F)** Quantification of the proportion of neurons expressing Tbr2 among the EGFP+/EdU+ population. **(G,H)** Analysis of the cell cycle exit index. IUE was performed at E13.5 with control shRNA or mIPO8 shRNA-2. Mice were injected i.p. with EdU 24 h before the sacrifice at E15.5. **(G)** Immunolabeling for EGFP (green), EdU (gray) and Ki67 (red), a proliferation marker. **(H)** Quantification of the cell cycle exit index (number of EGFP+/EdU+/Ki67− cells among the EGFP+/EdU+ population). The blue arrows show triple stained cells. The neurons were counted in the VZ/SVZ and IZ. VZ/SVZ, ventricular/subventricular zone; IZ, intermediate zone; CP, cortical plate; *p < 0.05, ***p < 0.001.

Hence, we proceeded to study the effect of *Ipo8* knockdown on the mitotic progression. Toward this purpose, time-mated dams were IUE with either control shRNA or mIPO8 shRNA-2 at E13.5 followed by injection with EdU 1 h before sacrifice at E15.5. The embryonic brains were analyzed to detect EGFP, EdU and PH3 (an M-phase marker). The mitotic index, that correspond to the proportion of EGFP cells double positive for EdU and PH3, was significantly increased upon knockdown (10.2 ± 1.9%; *p* < 0.05) compared to control shRNA (5 ± 1.1%; Figures 3A,B). This accumulation of mitotic cells suggests that *Ipo8* knockdown slowed down the M-phase progression of cortical progenitors.

While our ISH experiments show a clear signal in the entire VZ/SVZ region, which demonstrate that proliferative cells express *Ipo8* mRNA, the transcript was also detected by RNA-seq analysis within FACS-purified AP from E12 to E16 brains (L. Nguyen and G. Agirman, personal communication). The two different cortical progenitor pools can be distinguished based on the expression of specific markers i.e., Sox2 and Tbr2 for APs and BPs respectively (Sessa et al., 2008; De Juan Romero and Borrell, 2015). Hence, we quantified the proportion of EGFP and EdU double positive cells expressing Sox2 or Tbr2. The *Ipo8* knockdown produced a higher percentage of cells positive for Sox2 compared to controls (control shRNA: 84 ± 2%; mIPO8 shRNA-2: 90 ± 1%, *p* < 0.05; Figures 3C,D) with no variation for Tbr2 expressing cells (control shRNA: 42 ± 2%; mIPO8 shRNA-2: 45 ± 3%; *p* = 0.3; Figures 3E,F). These data suggest that AP cell population is specifically affected by *Ipo8* knockdown.

We next studied the cell cycle exit of APs in time-mated E13.5 embryos that were IUE with either control shRNA or mIPO8 shRNA-2 followed by i.p. injection of EdU 24 h before sacrifice at E15.5 to analyze EGFP, EdU and Ki67. Among EGFP+ electroporated cells, we monitored EdU+/Ki67+ cells that correspond to cycling cortical progenitors, as well as EdU+/Ki67− cells that are postmitotic neurons that have exited the cell cycle after EdU injection (Pacal and Bremner, 2012). We found that the fraction of Ki67− cells in the EGFP+/EdU+ population was significantly lower (*p* < 0.001) following knockdown (control shRNA: 78 ± 2%; mIPO8 shRNA-2: 64 ± 3%), suggesting that acute loss of IPO8 reduced the neuronal output from APs (Figures 3G,H).

Together, these data indicate that IPO8 regulates both division and cell cycle exit of neuronal progenitor cells, especially APs.

### Loss of IPO8 Expression in Projection Neurons Result in Migration Delay and Dendritogenesis Defects

**Figure 4:**
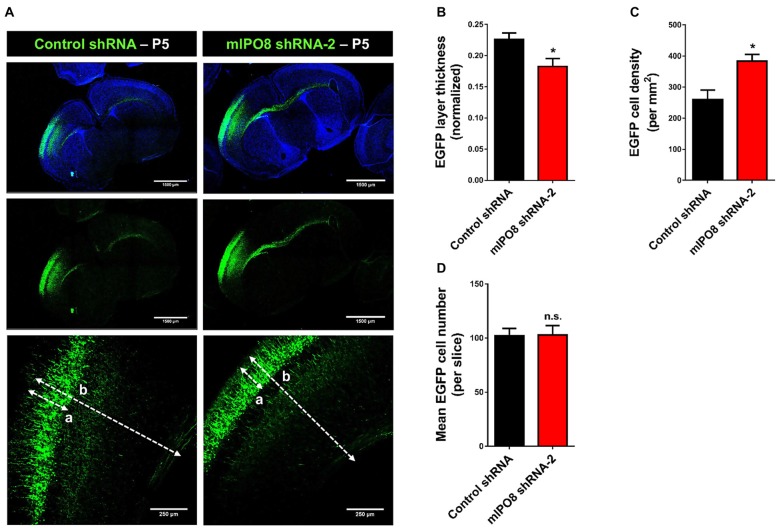
Long term *Ipo8* knockdown delayed radial migration of projection neurons. **(A)** Radial migration after long term *Ipo8* mRNA knockdown. Distribution of EGFP-positive cells at P5 i.e., 9 days after IUE at E14.5 of a plasmid co-expressing EGFP and either a control shRNA or mIPO8 shRNA-2. The dashed lanes “a” and “b” correspond to the thickness of the EGFP layer and of the cortex respectively. **(B–D)** Normalized thickness, mean number of neurons and neuronal density of the EGFP layer. The normalized layer thickness corresponds to the a/b ratio. *p < 0.05.

As IPO8 knockdown impaired early radial migration, we decided to evaluate its consequences at later developmental stages. After birth, the projection neurons have reached their final destination in the cortex and begin to establish and consolidate synaptic connections amongst themselves by P14–P15 (Hoshiba et al., 2016). Brains were subjected to IUE with control shRNA or mIPO8 shRNA-2 at E14.5 and analyzed at postnatal day P5. At this age, we observed that transfected neurons were appropriately localized to the upper region of the neocortex (Figure 4A). This indicates that radial migration of *Ipo8*-suppressed neurons was only delayed when analyzed at E17.5. However, we noticed that the relative thickness of the EGFP layer (i.e., EGFP thickness divided by entire cortical thickness) was ~20% thinner after IPO8 knockdown (*p* < 0.05; Figure 4B). This is due to a higher density of EGFP-neurons (*p* < 0.05; Figure 4C) as the total number of EGFP cell is similar in both conditions (Figure 4D). This increased neuronal density could result from a defect of dendrites development as demonstrated in Kawabe et al. (2010).

**Figure 5:**
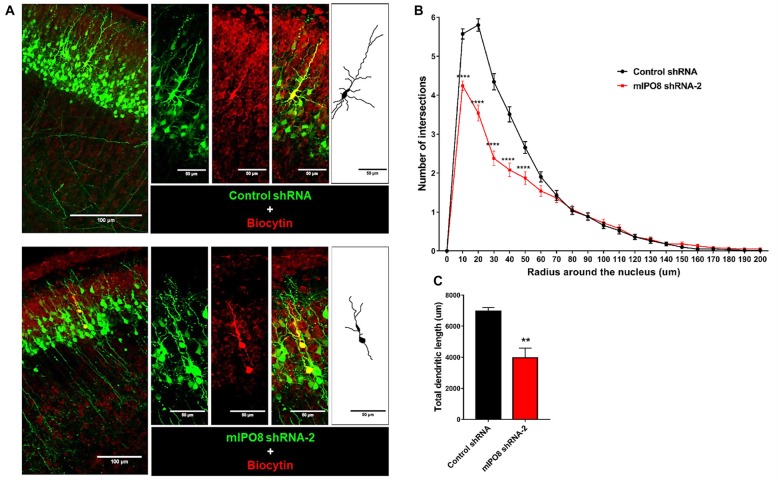
The dendrite tree of projection neurons at P5 is influenced by IPO8. **(A)** High magnification images and schematic drawing of EGFP expressing neurons filled with biocytin. The experimental conditions are the same as in Figure 4. **(B)** Scholl analysis of the dendritic complexity. **(C)** Total dendrite length of neurons. **p < 0.01, ****p < 0.0001.

**Figure 6:**
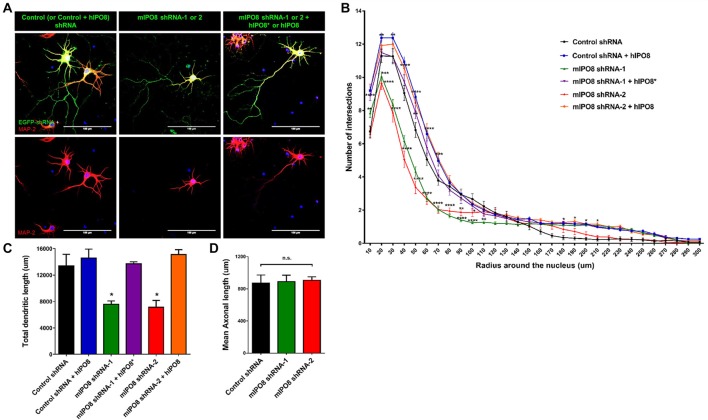
*In vitro* dendritogenesis of cortical neurons is impacted by *Ipo8* knockdown. **(A)** Immunolabeling for EGFP and Map2 of representative cortical neurons. IUE was performed at E13.5 with either shRNA producing plasmid alone for knockdown studies or with both the shRNA and hIPO8 producing plasmids for rescue experiments (see Figure 2). Two days later, cortices were dissociated and neurons were cultured for 7 days *in vitro* (DIV). **(B)** Sholl analysis of the dendritic complexity. **(C)** Total dendrite length of neurons. **(D)** Axonal length of neurons. The control shRNA condition was taken as reference for statistical analysis *p < 0.05, **p < 0.01, ***p < 0.001, ****p < 0.0001.

To investigate the role of IPO8 in dendritic development, we examined the morphology of the dendritic tree of neurons under both *in vivo* and *in vitro* experimental designs. In the former, Sholl analysis was performed after biocytin injection at P5 of neurons IUE at E14.5 whereas in the latter, analysis was done on cortical neurons cultured for 7 days *in vitro* (DIV) in the latter after IUE. Sholl analysis of individual EGFP-positive neurons *in vivo* revealed that *Ipo8* suppression led to less complex dendritic projections and a decreased total length (*p* < 0.01) compared to control neurons (Figure 5). Interestingly, their axons that form the callosal projections were not affected, as they reach the contralateral hemisphere in both conditions (Figure 4A). *In vitro*, the knockdown of *Ipo8* with either mIPO8 shRNA-1 or -2 also impaired the dendritic complexity and the total dendritic length of cortical neurons (*p* < 0.05 for both shRNA; Figure 6). Furthermore, the rescue experiment restored the total dendritic length default (*p* = 0.8 for mIPO8 shRNA-1 + hIPO8* and *p* = 0.9 for mIPO8 shRNA-1 + hIPO8 both compared to control shRNA) and most of the dendritic complexity (Figure 6). *In vitro*, the axonal length was not impacted by the knockdown (Figure 6D). In combination, our *in vivo* and *in vitro* data indicate that *Ipo8* knockdown causes a cell-specific defect on dendritic formation of projection neurons.

### IPO8 Is Required for the Migration of Interneurons

**Figure 7:**
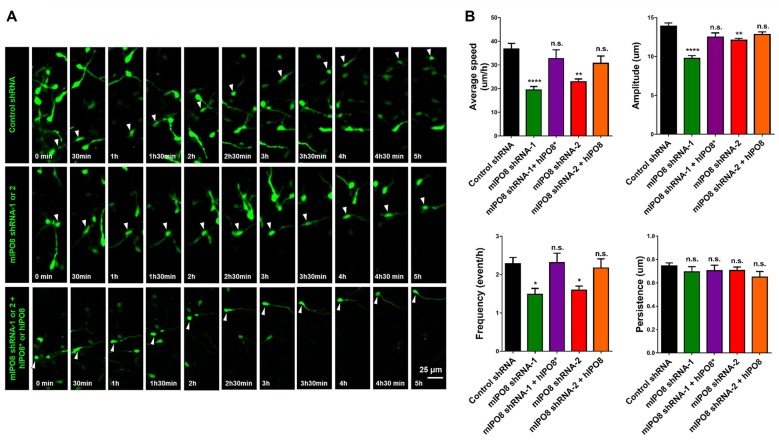
Interneuron migration is decreased by acute IPO8 knockdown. **(A)** Typical recordings of the movement of interneuron expressing control or mIPO8 shRNA. MGE explants electroporated by shRNA/EGFP producing plasmids were cultured on cortical feeders for 1 or 2 days and were recorded by live imaging microscope every 5 min during 5–10 h. **(B)** Quantification of the average speed, persistence, frequency and amplitude of interneurons. *p < 0.05, **p < 0.01, ****p < 0.0001. MGE: median ganglionic eminence.

As we showed a strong expression of *Ipo8* mRNA in the ganglionic eminences, its implication in the migration of interneurons was evaluated. After electroporation, MGE explants were cultured on cortical feeder and time lapse imaging of EGFP labeled interneurons migration was quantified (Figure 7A). Four parameters were analyzed: migration average speed, frequency of somal translocation, amplitude of somal translocation and directional persistence (measure of the efficiency of the movement in the space; Figure 7B). *Ipo8* knockdown led to a significant decrease of migration average speed from 37 ± 2.3 μm/h for control shRNA to 19 ± 1.5 μm/h for mIPO8 shRNA-1 (*p* < 0.0001) and 23 ± 1.2 μm/h for mIPO8 shRNA-2 (*p* < 0.01). Rescue restored migration average speed to 33 ± 3.7 μm/h for shRNA-1 (*p* = 0.6) and 31 ± 3.1 μm/h for shRNA-2 (*p* = 0.3). These changes correlated with a significant decrease of amplitude and frequency of somal translocation. Amplitude decreased from 14 ± 0.4 μm for control shRNA to 10 ± 0.5 μm for shRNA-1 (*p* < 0.0001) and 12 ± 0.2 μm for shRNA-2 (*p* < 0.01) but was rescued to 12.5 ± 0.5 (*p* > 0.5) and 13 ± 0.3 (*p* = 0.2). The frequency of somal translocation decreased from 2.3 ± 0.16 event/h for control shRNA to 1.5 ± 0.16 for mIPO8 shRNA-1 (*p* < 0.05) and 1.6 ± 0.1 event/h for mIPO8 shRNA-2 (*p* < 0.05) and was restored to 2.3 ± 0.25 and 2.2 ± 0.24 respectively when hIPO8 is overexpressed. The directional persistence of migration was not modified upon acute knockdown of IPO8 (*p* > 0.1). Altogether, our data indicate that *Ipo8* suppression impedes the migration of interneurons in the developing cerebral cortex.

## Discussion

The aim of this study was to investigate the role of IPO8 in brain development as it is an important member of the importin-β family, which is involved in the transport of several protein cargoes essential for corticogenesis.

First, we demonstrated a clear IPO8 expression in the MGE/LGE, the VZ/SVZ and the CP of embryonic mouse brain, suggesting this importin-β possesses important roles for the physiology of neural progenitors and postmitotic differentiating neurons.

As we found an expression in the VZ/SVZ and MGE/LGE, we investigated the implication of IPO8 in radial and tangential migration of neuroblasts. Acute IPO8 knockdown with two different shRNA in nascent apical progenitor projection neurons impairs their division. Acute IPO8 knockdown in MGE explants impairs the migration of interneurons. These phenotypes are cell-specific as the overexpression of human IPO8 protein could completely rescue the effect of both shRNA in these two developmental steps.

Regulated synthesis of specific karyopherins has been shown to mediate cell fate outcomes in embryonic stem cell systems (Yasuhara et al., 2007; Young et al., 2011). Therefore, we analyzed the role of IPO8 in projection progenitor cell neurogenesis. The knockdown of IPO8 increases the cell cycle length and probably maintains progenitors in a proliferative state as the cell cycle exit was decreased. As a consequence, we observed an increase in the percentage of apical Sox2 but not basal Tbr2 neural progenitor cell population. Note that the sum of both percentages is higher than 100% because a minority of the differentiating progenitors continue to express Sox2 while beginning to express Tbr2 (Englund et al., 2005; Sessa et al., 2008).

Interestingly, at P5, *Ipo8*-suppressed neurons reached the cortical upper layers in proportion that is comparable to the control. However, their dendritic tree is much less complex whereas axons seem unaffected. Cortical neuronal culture *in vitro* confirmed that IPO8 knockdown changes the growth of the dendritic arbor. Altogether, our data indicate that IPO8 plays distinct roles in controlling neuronal production and dendritic branching during early brain development.

Also, radial migration process seems impacted when observed after 3 days, projection neurons finally could reach their cortical layers in the same proportion as in the control condition. There are numerous examples showing that, when radial migration is altered at short term, a large proportion of neurons will not reach the cortical upper layers. Knockdown of Doublecortin (Dcx) in the embryo using shRNA produces a subcortical band heterotopia beneath normotopic cortex in the adult brain (Bai et al., 2003). Knockdown of Lis-1 or Dab2ip, a Ras GTPase-activating protein, also induces such migration defect and many neurons remains in the lower cortical layers at postnatal ages (Tsai et al., 2005; Lee et al., 2012). However, the knockdown of the 14-3-3 gamma protein affects the morphology and the average speed of migrating neurons but finally, it only delayed their migration as they could reach their expected layer in the CP (Wachi et al., 2016). A compensatory mechanism due to functional redundancy could explain our results. Indeed, karyopherins are flexible proteins that show overlapping as well as distinct cargoes specificities (Forwood et al., 2010). This is even more likely to happen between IPO8 and IPO7 as they are close paralogs with 67% identity as compared to the 10%–20% overall identity within the importin-β family (Quan et al., 2008). Some IPO8 cargoes, such as Dicer (Doyle et al., 2013), FOXO3 (Putker et al., 2015) or Smad4 (Yao et al., 2008), have also been reported to bind IPO7. In addition, with some exceptions, karyopherins are relatively well expressed during mouse development and in most cell type of the adult brain (Quan et al., 2008; Zhang et al., 2014). For example, importin-α5 has been shown to be necessary for neuronal differentiation *in vitro*, however, no gross abnormalities have been observed in the brain of knockout mice because of an increased expression of importin-α4 that probably compensate its deficit (Shmidt et al., 2007). However, qRT-PCR experiments did not reveal any changes in IPO7 mRNA expression (data not showed) but compensation may take place only at the protein level. Alternatively, we cannot exclude that the remaining residual amount of IPO8 is sufficient to transport smaller amounts of cargo, so that the migration process is slowed down but not blocked.

Importantly, the dendrite size and complexity of the neurons is decreased when IPO8 is knocked down. This does not appear to be the consequence of the “migration delay”, which in turn, could have delayed the differentiation step. Indeed, the length of the axons forming the callosal projections is not affected, and *in vitro* experiments produced similar changes. Dendritogenesis is regulated by intrinsic and extrinsic cues that are intimately linked (Puram and Bonni, 2013). Our defects are relatively similar to those described at P4 in early differentiating neurons after Cux2 knockdown (Cubelos et al., 2010). Therefore, they suggest IPO8 controls an early intrinsic factor that is necessary for dendrite development. It will be interesting to perform longer-term experiments to analyze spine morphology/numbers as well as record their synaptic activity.

Among the IPO8 cargoes proposed in the literature, several are implicated in brain development. As co-Smad, Smad4 is necessary for both TGF-β and BMP signaling pathways. So IPO8 knockdown should decrease the efficiency of the signal transmitted to the target genes. Both pathways activate several target genes such as Id1 or Crmp2 for example, that are involved in different brain development steps such as the precocious differentiation of cortical progenitors or the development of dendrites (Ross et al., 2003; Sun et al., 2010; Makihara et al., 2016). Dicer and Ago2 are two other IPO8 cargoes and it was shown that IPO8 is required for nuclear localization of Ago2 and miRNA gene silencing (Weinmann et al., 2009). Both cargoes are part of the machinery that catalyzes gene silencing at the posttranscriptional and translational level (Ross and Kassir, 2014). Dicer is necessary for appropriate specification of radial glia during early development, for cortical neurogenesis but also dendritogenesis, axonogenesis and synapse formation (Nowakowski et al., 2013; Zhang et al., 2013). Finally, eIF4E is the component of the translation machinery that binds the (m^7^G)-cap of mRNA (Volpon et al., 2016). It exports particular mRNA containing 4E-SE elements but this export can be decoupled from translation (Culjkovic et al., 2006). The knockdown of IPO8 in U2OS cells changed the nuclear localization of eIF4E and decreased the translation of specific proteins such as Bcl-1, Mcl-1 and c-Myc for example (Volpon et al., 2016). In the developing brain, eIF4E mRNA is enriched in the VZ and CP (DeBoer et al., 2013). An up-regulation of cap-dependent mRNA translation via mTORC1 signaling or the knockdown of 4E-BP, a protein that completes with eIF4E for (m^7^G)-cap, induces the misplacement of cortical neurons (Lin et al., 2016).

Interestingly Ago2, Dicer and eIF4E are components of the P-bodies (Processing bodies). These P-bodies are cellular granules found in the cytoplasm but also in dendrites (Oh et al., 2013). They are most probably sites of mRNA storage, reversible mRNA repression and mRNA decay. They are important for the regulation of the “mRNA cell cycle” and for local translation in neurons and so, IPO8 could regulate these processes (Olszewska et al., 2012). This putative regulation of the mRNA cell cycle is interesting as a multitude of molecular pathways are necessary for a cell to progress from neural progenitor stage to the mature neuron. Moreover, the transcriptional programs evolve during the progression of cells between the different zones of the embryonic brain (Ayoub et al., 2011). Given the wide range of possible IPO8 actions that can explain our results, it will be important in the future to develop a mouse line that allow acute conditional inactivation of IPO8 in forebrain in order to evaluate neurogenesis and corticogenesis (i.e., neuronal production and fate, laminar positioning) and to analyze the consequences of IPO8 knockdown/knockout on the physiology of the different cell types that arise during brain development. Selection of cells at different time points using FACS and subsequent analysis of their transcriptome and/or proteome would be the method of choice.

In summary, we report for the first time that IPO8 plays a role in neuronal migration and dendrite extension in the developing mammalian neocortex. These findings lay the foundation for further studies aiming to elucidate the molecular mechanisms of IPO8 activity in the developing brain. Indeed, in the cerebral cortex, defects in neuronal precursor proliferation, morphogenesis, cell migration, dendritogenesis and synaptogenesis can lead to mental retardation, autism spectrum disorders, epilepsy or other neurodevelopmental brain disorders. Therefore, elucidating the pathways that integrate cellular cues implicated in brain development, are essential to understand cortical architecture and connectivity.

## Author Contributions

GN performed most of the experiments. BC performed cloning, DE biocytin injections and IG-N some IUE. CS and LN taught and/or gave input for the design of migration experiments. TG, LdN and BL supervised the work. GN, LdN, IG-N and BL wrote the article with critical advices from AD-E, MT, LN and TG.

## Conflict of Interest Statement

The authors declare that the research was conducted in the absence of any commercial or financial relationships that could be construed as a potential conflict of interest.
