# Supplementary material for: Importin-8 Modulates Division of Apical Progenitors, Dendritogenesis and Tangential Migration During Development of Mouse Cortex
Source: Front Mol Neurosci. 2018 Jul 10;11:234. doi: 10.3389/fnmol.2018.00234 (PMC6048241; doi:10.3389/fnmol.2018.00234)
Supplement: Supplementary file 1 [file Data_Sheet_1.PDF]

Supplementary data

A

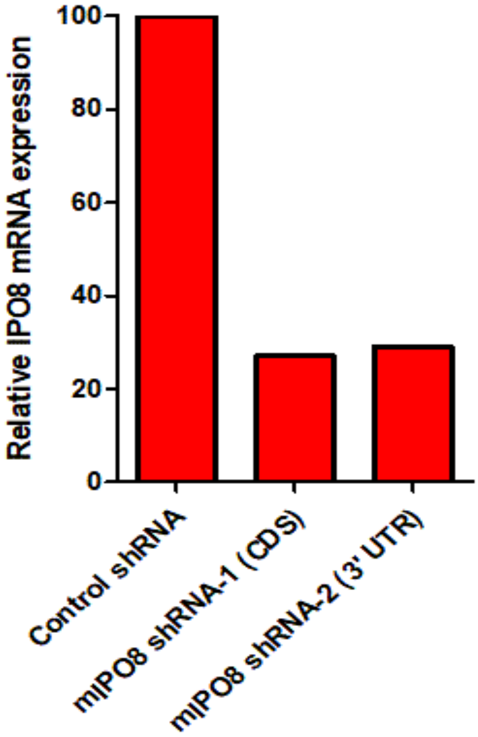

B

shRNA	Sequence (5'-3')
**Control shRNA** (Luciferase)	TACGCGCATAAGATTAGGG**TTCAAGAGA**CCCTAATCTTATGCGCGTA
**mIPO8 shRNA-1** (3070-3090)	GGACAACAGTGTGGATGAATA**TTCAAGAGA**TATTCATCCACACTGTTGTCC
**mIPO8 shRNA-2** (3805-3825)	GCATCCTTGCTTCCTTCAACC**TTCAAGAGA**GGTTGAAGGAAGCAAGGATGC

Figure S1. Efficiency (A) and sequence (B) of the two shRNA directed against mIPO8 that were selected. qRT-PCR was performed on FACS selected Neuro-2A cells to evaluate the efficiency of the shRNAs. The position of the targeted sequence on the mRNA (NM_001081113) is given and the nucleotides forming the loop are in bold.

**mIPO8 shRNA-1**	G GAC AAC AGT GTG GAT GAA TA
	L D N S V D E Y
**hIPO8***	CT**T** GA**T** AA**T** **TCC** GT**A** GA**C** GA**G** TAT

Figure S2. Nucleotide sequences of mouse IPO8 targeted by shRNA-1 and of the mutated human IPO8 transcript (hIPO8*) generated to make it resistant to shRNA-1. The nucleotides in bold and underlined differ between the mutated human and wild-type mouse sequences. The amino acids they encode are identical.

**mIPO8-F**	CTTCCTGGGACACCATGACC	2701-2720	(overlap exons 21-22)
**mIPO8-R**	GACATGCGCCGATACACAAC	3583-3564	(exon 25)

Figure S3. Sequences of the primers used for RT-PCR, their location along the mRNA (NM_001081113) and corresponding exon number.

**mIPO8-F**	CGTGACAACATTGTGGAAGG	497-516	(exon 3)
**mIPO8-R**	CCAGTGACCAGGAAAATCGT	601-582	(exon 4)
**mIPO7-F**	TCAGAACAACTGGATTTACCTGTG	133-156	(exon 2)
**mIPO7-R**	CGATCAGGCCAATATTGTGTTA	212-191	(exon 3)

Figure S4. Sequences of the primers used for qRT-PCR, their location along the mRNA and corresponding exon number (Shimada et al., 2009; Sangel et al., 2014)**.**

Supplementary data References

Sangel P, Oka M, Yoneda Y (2014) The role of importin-ßs in the maintenance and lineage commitment of mouse embryonic stem cells. *FEBS Open Bio*, 4, 112-20

Shimada I, Matsui K, Iida R, Tsubota E, Matsuki T. (2009). Time course of housekeeping gene expression changes in diffuse alveolar damage induced by hyperoxia exposure in mice. *Leg. Med.* 11, S151–S154
